# Relationship between Nordic hamstring strength and maximal voluntary eccentric, concentric and isometric knee flexion torque

**DOI:** 10.1371/journal.pone.0264465

**Published:** 2022-02-25

**Authors:** Satoru Nishida, Masatoshi Nakamura, Ryosuke Kiyono, Shigeru Sato, Koki Yasaka, Riku Yoshida, Kazunori Nosaka

**Affiliations:** 1 Faculty of Sports and Health Science, Fukuoka University, Fukuoka, Japan; 2 Institute for Human Movement and Medical Sciences, Niigata University of Health and Welfare, Niigata, Japan; 3 Department of Physical Therapy, Niigata University of Health and Welfare, Niigata, Japan; 4 Center for Exercise and Sports Science Research, School of Medical and Health Sciences, Edith Cowan University, Joondalup, Western Australia, Australia; Federation University Australia, AUSTRALIA

## Abstract

Nordic hamstring exercise is performed to prevent knee flexor muscle strain injuries and used to assess their injury risks. However, what exactly Nordic hamstring strength indicates is not clear. We investigated the relationship between Nordic hamstring strength and maximal voluntary contraction (MVC) torque of the knee flexors measured by an isokinetic dynamometer. Sixteen healthy young men who had not experienced hamstring strain injuries participated in the study. In Nordic hamstring, each participant was instructed to lean forward as far as possible in 3 s (approximately 30°/s), and force at the ankle joint of the dominant leg was measured during the movement. The force was multiplied by lower leg length and converted into torque. MVC torque of the knee flexors was measured isometrically at 30°, 45°, 60°, and 90° knee flexion joint angles, and concentrically and eccentrically at 30°/s and 60°/s in 10°–90° knee flexion for the dominant leg in a prone position. Correlations among the dependent variables were assessed using Pearson’s correlation coefficients. Peak Nordic hamstring torque ranged 96.8–163.5 Nm, and peak MVC eccentric torque ranged 50.7–109.4 Nm at 30°/s and 59.2–121.2 Nm at 60°/s. No significant correlations were evident between the peak Nordic hamstring torque and peak eccentric knee flexion torque (r = 0.24–0.3, p = 0.26–0.4). This was also the case for the Nordic hamstring torque and MVC torque of isometric (r = −0.03–0.1, p = 0.71–0.92) and concentric contractions (r = 0.28–0.49, p = 0.053–0.29). These results show that Nordic hamstring strength is not associated with the knee flexor torque measured by an isokinetic dynamometer. It may be that other factors than static and dynamic hamstring strengths affect Nordic hamstring strength.

## Introduction

Hamstring strain injuries are common in sports, consisting of 12–15% of all injuries [1]. The etiology of the hamstring strain injuries has been investigated in many studies, and risk factors and prevention strategies have been documented [2, 3]. However, it does not appear that the number of hamstring strain injuries has decreased significantly in the last 10 years [4, 5]. Since the knee flexors perform eccentric contractions to absorb force in knee extension movements to decelerate the momentum during the late swing phase in sprinting, hamstring strength, especially in eccentric contractions, is important to prevent its strain injuries [6, 7].

Eccentric knee flexor strength is commonly measured using an isokinetic dynamometer, which is considered to be a gold standard [8, 9]. Green et al. [8] reviewed the articles that examined the relationship between isometric, concentric and eccentric knee flexion torque and the risk of hamstring strain injuries, and showed that only eccentric knee flexor torque at a slower angular velocity (60°/s) could predict the risk of hamstring strain injuries. However, they stated that the isokinetic testing might not be suited for hamstring strain injury risk assessment, because the movements of the musculotendinous unit in sprinting are different from the measurement set-up in an isokinetic dynamometer. The authors suggested the necessity of alternative strength tests to assess eccentric strength that could better predict hamstring strain injury risks.

Some devices to assess Nordic hamstring (NH) strength are available, which have been used to predict hamstring strain injury risks [10, 11]. NH exercise is also often performed as a preventative measure of hamstring strain injuries [12] with the assumption that it can increase eccentric strength of the knee flexors [13]. It is also generally believed that NH strength represents eccentric strength of the knee flexors [14]. Mjølsnes et al. [14] reported that progressive NH training performed one to three sessions per week for 10 weeks effectively increased maximal eccentric knee flexion torque measured by an isokinetic dynamometer by 11% in well-trained soccer players. However, the relationship between NH strength and knee flexor strength measured by an isokinetic dynamometer has not been investigated by many studies.

To the best of our knowledge, only two studies have attempted to investigate the relationship between eccentric knee flexor strength measured by an isokinetic dynamometer and NH strength [15, 16]. van Dyk et al. [15] reported a poor correlation (r = 0.35) between NH force and eccentric knee flexion torque measured at 60°/s in a seated position among 337 professional male football players. The authors concluded that the low correlation between the two measurements was due to the differences in the measurement position such that sitting for the isokinetic measure and kneeling for the NH measure. Wiesinger et al. [16] used a supine position for isokinetic (30°/s) eccentric knee flexion torque measure and NH torque measured at the angular velocity of 30°/s among 25 healthy male student athletes, and reported a significant correlation between them (r = 0.58). However, they also showed an average difference of 19 Nm between the NH torque and the eccentric knee flexion torque, and stated that the difference could be due to the hip position and movement velocity differences. Thus, it appears that further studies are required to clarify whether NH strength indicates knee flexor eccentric strength. It should be also noted that the two previous studies did not include isometric and concentric knee flexor torque measures. It might be that NH strength is more associated with isometric than eccentric knee flexor strength.

Therefore, the present study examined the relationship between the NH strength and maximal voluntary contraction torque of the knee flexors measured by an isokinetic dynamometer during isometric, concentric, and eccentric contractions in a prone position. We hypothesized that isometric and eccentric knee flexion torque would be significantly correlated with the NH strength.

## Materials and methods

### Participants

Sixteen healthy male university students who habitually performed resistance training including that for the knee flexors 2–3 times a week, were recruited for the present study. Since the main purpose of this study was to examine correlations between NH strength and knee flexor maximal voluntary contraction torque assessed by an isokinetic dynamometer, a priori sample size was not calculated in this study. We assumed that the sample size (n = 16) was adequate to achieve the main purpose, because a previous study in which NH strength was examined [17] also used a similar sample size to that of the present study. Their mean ± SD (range) age, body mass, height and lower leg length were 21.4 ± 1.0 (21–24) years, 66.5 ± 4.6 (60.7–75.8) kg, 1.71 ± 0.05 (1.66–1.83) m, and 0.40 ± 0.03 (0.36–0.45) m, respectively. None of the participants had experienced hamstring strain injuries, knee joint injuries and low back pain. This study was approved by the institutional ethics committee and was conducted in conformity with the principles of the Declaration of Helsinki. Study procedures and potential risks were explained to the participants, and each participant provided a written informed consent before participation in the study.

### Experimental design

The participants reported to the laboratory on two separate days with a week between visits. The first visit was a familiarization session to practice NH (3 sets of 3 repetitions). In the second visit, NH strength and knee flexor maximal voluntary contraction (MVC) torque measures were performed. Before the NH strength measures, participants performed a warm-up exercise consisting of 5-min cycling on a stationary exercise bike (AFB6215, ALINCO, Japan) at 70–80 rpm (100 W), 10 repetitions of deadlift using a flywheel machine (kBOX4 Lite Advanced System, Exxentric AB, Stockholm, Sweden), and 3 sets of 5 split jumps. After the warm-up exercise, participants performed the NH strength test explained below. After the NH strength test, MVC torque of the knee flexors was measured using an isokinetic dynamometer as described below, with more than a 10-min rest between the NH strength and MVC torque measures. The MVC torque measures were performed after the NH strength test, since we thought that it was better to test the NH strength in a fresher condition, although the NH strength test could have affected the MVC torque measures.

### Nordic hamstring (NH) strength

**Fig 1:**
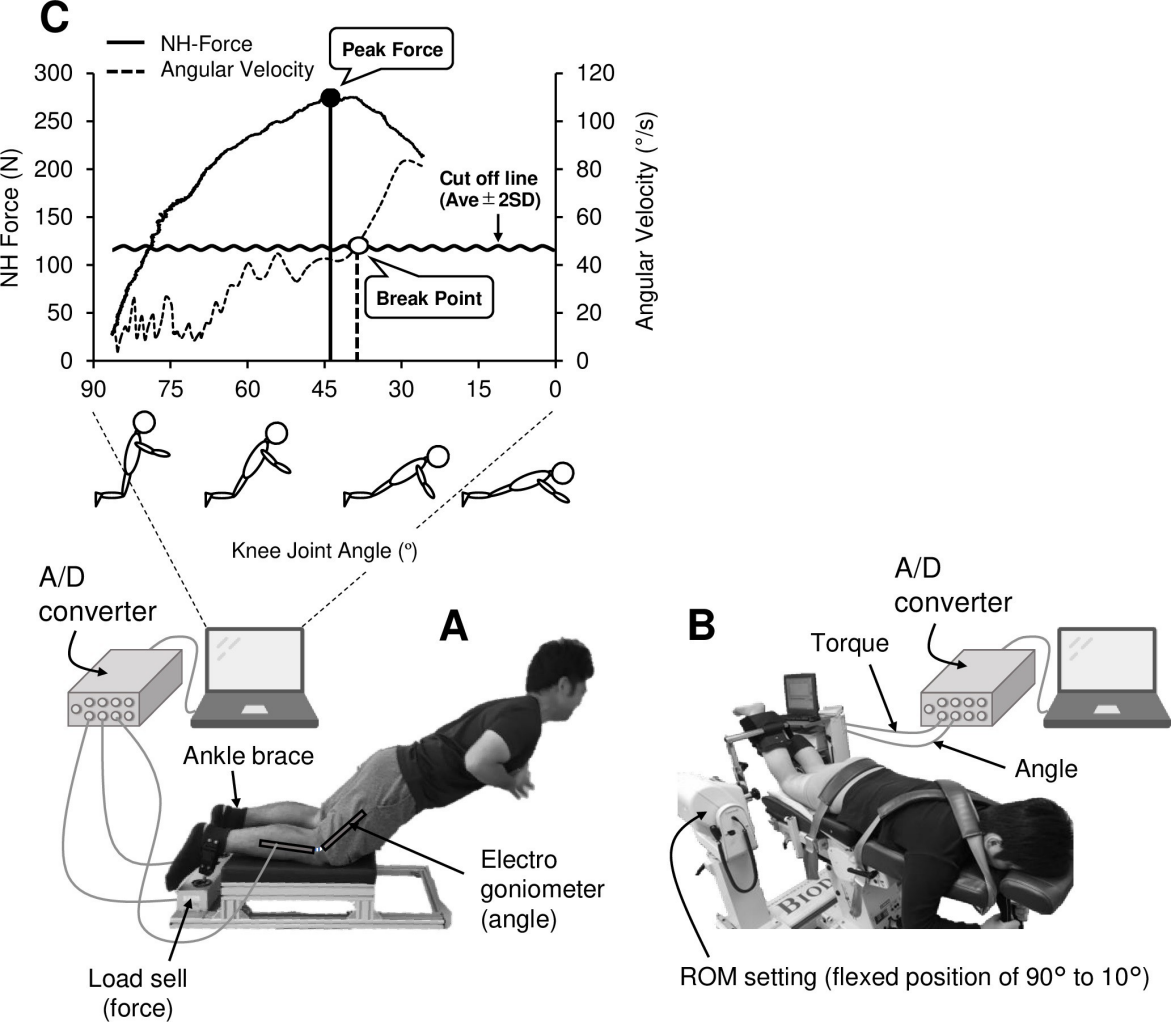
Measurement setup for the Nordic hamstring strength (A), and maximal voluntary contraction (MVC) torque measurements with an isokinetic dynamometer (B). An example of recording of Nordic hamstring force measure with angular velocity is shown in C, in which how the peak force and break point were determined are shown. Reprint from the original image under a CC BY license, with permission from Masatoshi Nakamura and Kazuya Yoshida (February 2021).

The setup for the NH strength measure is shown in Fig 1A. Each participant was in a kneeling position on a custom-made NH device, with each ankle being secured above the lateral malleolus by an ankle brace that was attached to a load cell. The force against the ankle brace in the vertical direction was measured by the load cell connected to a PowerLab16/35 (AD Instruments, Bella Vista, Australia). The force was calibrated before each test using a known weight that was hung from the ankle brace, with the device being set up upside down. A single-axis electro-goniometer (FA-DL-260, 4assist, Japan) was attached to the lateral aspect of the right knee to monitor the knee joint angle during the NH. Each participant was instructed to gradually lean forward from the initial kneeling position at 90° knee flexion to a prone position in 3 s with the arms crossed at the chest and the hip joint being at a full extension. To standardize the velocity of the movement, the participants were instructed to lean forward with a constant angular velocity indicated by a metronome as much as possible. In the present study, the participants were asked to perform the NH trial three times to assess reliability of the measure and choose the best attempt with the highest peak NH force. Force and angle data were transferred from the PowerLab16/35 to a personal computer (VersaPro, NEC, Japan) at 1000 Hz. As illustrated in Fig 1C, peak force, angle at peak force, break point angle, force at break point angle and kinematic parameters (i.e., range of motion, average angular velocity) were analyzed from the data obtained from the system. The break point angle was considered to represent the ability to control the NH movement. Previous studies defined the break point angle as the point which the knee angular velocity was greater than 10°/s during the NH at the slowest possible knee angular velocity [18, 19]. In the present study, the angular velocity was set at approximately 30°/s, thus it was necessary to set a different criteria for the break point angle. We defined the break point angle as the knee joint angle at the first instance of a velocity that exceed the average ± 2SD lean forward angular velocity. When comparing to the MVC torque of the knee extensors, NH force was multiplied by lower leg length and converted into torque.

### MVC torque of the knee flexors

Each participant performed several MVC measures on a Biodex System 3 (Biodex Medical Systems, Shirley, NY, USA). The measures were performed in a prone position (Fig 1B) to make it closer to the position of the NH strength measure. Each participant was secured in the prone position, and the upper back region and pelvis were stabilized using Velcro straps. The axis of rotation of the dynamometer lever arm was aligned with the lateral epicondyle of the femur. The range of motion was set between the flexed position of 90° and 10° (0° = full knee extension) for both concentric (10° to 90°) and eccentric contractions (90° to 10°). Participants performed warm-up trials at ~80% of subjectively perceived maximum effort twice before the isometric torque measures at 90, 60, 45, and 30° knee flexion, respectively. They also performed three concentric and eccentric contractions at ~80% of subjectively perceived maximum effort for the range of motion at the angular velocity of 60°/s and 30°/s, respectively before concentric and eccentric torque measures. MVC torque in isometric was measured twice at 90, 60, 45, and 30° knee flexion in this order. Subsequently, concentric MVC torque was measured three times followed by eccentric MVC torque at an angular velocity of 60°/s and 30°/s in this order, respectively. These test trials were conducted with a 30-s rest between contractions and a 3-minute rest between different modes. Participants were verbally encouraged by the investigator to generate maximal force for the entire range of motion for concentric and eccentric contractions. As explained for the NH strength measure, the torque and knee angle data were transferred to a personal computer at 1000 Hz by the PowerLab system. The data that showed the highest torque for each measure were used for further analyses.

### Statistical analyses

Shapiro Wilk test was used to assess the normality of the measures. NH parameters (i.e., peak NH force and NH torque, peak force angle, break point angle, force and torque at break point angle) derived from three trials were compared using repeated-measures analysis of variance (ANOVA), and its effect size (ES) was obtained by calculating partial eta squared (*η*^*2*^). According to Richardson [20], *η*^*2*^ is classified as small (0.01–0.059), moderate (0.06–0.137) and large (≥0.138). Relative and absolute reliability were assessed by the intraclass correlation coefficient (ICC_1,1_), standard error of measurement (SEM) and coefficient of variation (CV). The ICC of 0.90 or greater was regarded as high, between 0.80 and 0.89 as moderate, and 0.79 or less as poor [21], and CV of 10% or less was considered reliable [22]. Pearson’s correlation coefficients were used to examine the relationships between torque and angle-related variables from the NH strength measures and those from the MVC torque measures. Statistical significance was set at *P* < 0.05. Statistical analyses were performed using SPSS software version 27 (SPSS Inc, Chicago, IL).

## Results

### Force, torque, angle and kinematic parameters during NH

Peak NH force, peak NH torque, and the angle at peak force were 325.6 ± 45.3 N (range: 217.6–389.3 N), 130 ± 17.6 Nm (range: 96.8–163.5 Nm) and 32.5 ± 14.9° (range: 6.4–52.5°), respectively. Although the break point angle (32.0 ± 9.7°, range: 14.1–48.9°) was not significantly different from the angle at peak force, the force at the break point angle (297.4 ± 43.3 N, range: 216–376.5 N) and torque at the break point angle (112.4 ± 35.1 Nm, range: 87.5–158.1 Nm) were significantly smaller than the peak NH force and peak NH torque. During NH, the range of motion was 75.5 ± 12.2° (range: 50–90°), and its average lean forward angular velocity was 28.8 ± 6.4°/s (19.2–41.3°/s).

### Reliability of NH parameters

Table 1 presents the variables in NH strength measures over three trials, and their reliability parameters such as ICC_1,1_, SEM, and CV with a 95% confidence interval (95%CI). No significant differences among the three trials were found for peak NH force (*η*^*2*^ = 0.005), peak NH torque (*η*^*2*^ = 0.005), angle at peak force (*η*^*2*^ = 0.003), break point angle (*η*^*2*^ = 0.07), force at break point (*η*^*2*^ = 0.002) and torque at break point (*η*^*2*^ = 0.002). The relative reliability was moderate for peak NH force (ICC = 0.83, CV = 2.7%), peak NH torque (ICC = 0.83, CV = 2.7%) and angle at peak force (ICC = 0.86, CV = 8.3%); however, the other parameters did not show high relative reliability (break point angle: ICC = 0.54, CV = 8.4%; force at break point angle: ICC = 0.46, CV = 10.0%; torque at break point: ICC = 0.48, CV = 9.9%).

**Table 1:** Nordic hamstring peak force and peak torque, angle at peak force, break point angle, and force and torque at break point angle (mean ± SD, range of 16 participants) over three trials (Test 1 –Test 3) for intraclass correlation coefficient (ICC), standard error of measurement (SEM) and coefficient of variation (CV) with 95% confidence interval (95%CI).

	Test 1 (Min—Max)			Test 2 (Min—Max)			Test 3 (Min—Max)			ICC (95%CI)	SEM (95%CI)	CV (95%CI)
**Peak Force (N)**	303.6	±	54.6	302.3	±	53.7	310.6	±	51.2	0.83 (0.63–0.94)	7.8 (4.7–11.0)	2.7 (1.6–3.8)
	(212.6–377.3)			(185.5–388.4)			(200.2–389.3)					
**Peak Torque (Nm)**	122	±	21.2	121.5	±	21.5	124.8	±	20.3	0.82 (0.61–0.93)	3.3 (1.9–4.6)	2.7 (1.6–3.9)
	(93.5–158.5)			(82.5–162.9)			(89.1–163.5)					
**Angle @ Peak force (°)**	34.4	±	15.3	36.2	±	15.7	34.2	±	17.8	0.86 (0.69–0.95)	1.8 (0.8–2.8)	8.3 (1.6–15.0)
	(4.5–52.5)			(1.7–59.2)			(6.4–55.3)					
**Break Point Angle (°)**	37.2	±	7.0	35.2	±	8.0	31.7	±	9.6	0.54 (0.21–0.81)	2.8 (0.8–4.7)	8.4 (2.4–14.3)
	(26.8–48.9)			(23.3–46.8)			(14.1–44.0)					
**Force @ Break Point Angle (N)**	263.1	±	58.6	268	±	47.9	269.4	±	62.8	0.46 (0.12–0.76)	23.4 (11.5–35.2)	10.1 (4.9–15.3)
	(131.4–336.6)			(148.8–336.9)			(137.8–376.5)					
**Torque @ Break Point Angle (Nm)**	105.9	±	23.8	107.7	±	19.3	112.4	±	35.1	0.47 (0.15–0.78)	10.1 (5.6–14.6)	10.0 (4.8–15.2)
	(51.9–138.9)			(66.2–138.1)			(87.5–158.1)					

### MVC torque of the knee flexors during isometric, concentric and eccentric contractions

Table 2 shows MVC torque during isometric, concentric and eccentric contractions, and peak torque angle in the concentric and eccentric contractions. Peak MVC torque in isometric knee contraction was the largest at 30° (75.5 ± 14.5 Nm) followed by 45° (71.4 ± 15.9 Nm), 60° (68.1 ± 16.2 Nm), and 90° (55.5 ± 14.1 Nm). The peak MVC torque in eccentric knee flexion at 30°/s (87.9 ± 14.6 Nm) was greater (p<0.05) than the peak MVC torques in isometric contractions at four knee joint angles. This was also the case for the peak MVC eccentric torque at 60°/s (84.5 ± 16.9 Nm) when compared with the peak MVC torques in isometric contractions at 45°, 60°, and 90° (p<0.05), but not at 30° (p = 0.13). The peak eccentric MVC torque was larger (p = 0.04) than the peak concentric MVC torque at 60°/s (76.1 ± 11.6 Nm) but similar (p = 0.22) to the peak concentric MVC at 30°/s (80.8 ± 13.3 Nm). The angle at peak torque in concentric knee flexion was smaller (p = 0.01) than that in eccentric knee flexion at 60°/s (21.5 ± 4.0° vs. 29.9 ± 9.7°), but not (p = 0.1) at 30°/s (19.5 ± 2.7° vs. 23.1 ± 8.2°).

**Table 2:** Maximal voluntary contraction torque of the knee flexors (mean ± SD, range of 16 participants) during isometric (30°, 45°, 60°, 90° knee flexion), concentric and eccentric (angular velocity of 30°/s, 60°/s) contractions, and the angle at peak torque in the isokinetic concentric and eccentric contractions.

		Mean ± SD			Range (Min − Max)		
**Peak Torque (Nm)**							
**Isometric**	30°	75.5	±	14.5	52.4	−	109.4
	45°	71.4	±	15.9	51.2	−	101.9
	60°	68.1	±	16.2	48.9	−	104.2
	90°	55.5	±	14.1	39.3	−	85.4
**Concentric**	30°/s	80.8	±	13.3	58.7	−	99.4
	60°/s	76.1	±	11.6	51.7	−	91.2
**Eccentric**	30°/s	87.9	±	14.6	50.7	−	109.4
	60°/s	84.5	±	16.9	59.2	−	121.2
**Angle @ Peak Torque (°)**							
**Concentric**	30°/s	19.5	±	2.7	16.2	−	25.3
	60°/s	21.5	±	4.0	16.7	−	29.7
**Eccentric**	30°/s	23.1	±	8.2	13.1	−	40.3
	60°/s	29.9	±	9.7	18.5	−	45.4

### Correlation among the variables

None of the physical characteristics were significantly correlated with peak NH force (height: r = −0.27, p = 0.3; body mass: r = 0.16, p = 0.6) and peak NH torque (height: r = −0.18, p = 0.95; body mass: r = 0.31, p = 0.25). Similarly, no significant correlations between the physical characteristics and peak MVC torque in isometric knee flexion at each angle and in concentric and eccentric knee flexion at each angular velocity were evident. However, a weak but significant correlation was found between peak concentric MVC torque at 60°/s and body mass (r = 0.52, p = 0.04). Within the NH parameters, peak NH torque had a significant correlation with NH force at break point angle (r = −0.8, p<0.01), but not with the angle at peak force (r = −0.36, p = 0.18).

**Fig 2:**
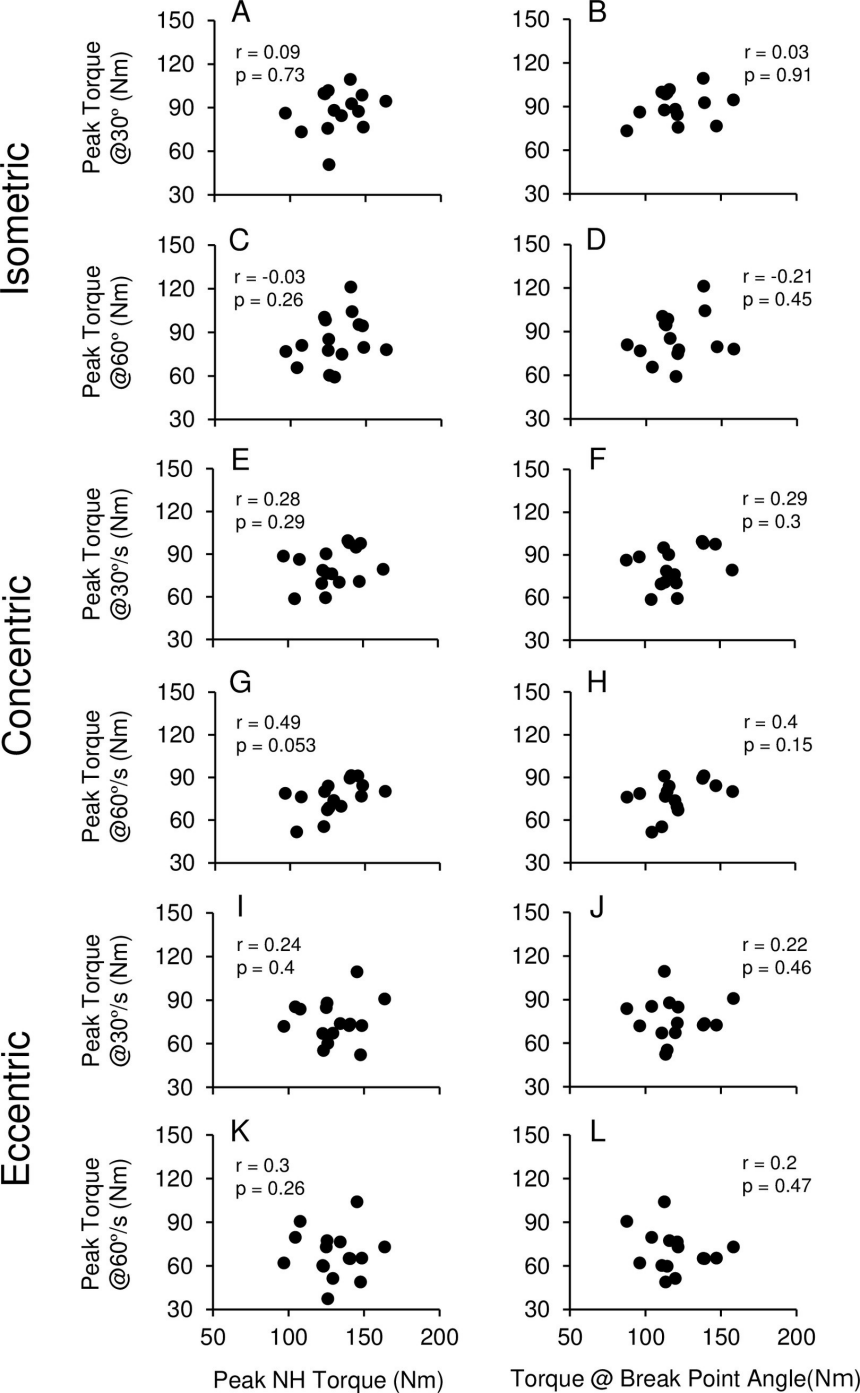
Correlations between peak Nordic hamstring torque (A, C, E, G, I, K) or Nordic hamstring torque at break point angle (B, D, F, H, J, L) and peak torque of knee flexors in maximal voluntary isometric (30°, 60°), concentric and eccentric contractions (30°/s, 60°/s) among 16 study participants. r and p values of Pearson correlation coefficient are shown in each figure.

Fig 2 shows correlations between NH torque parameters and some of the MVC torque measures. No significant correlations were evident between peak NH torque and peak knee flexion MVC torque in isometric contractions at 30°and 60° (Fig 2A and 2C) and other angles (45°: r = 0.08, p = 0.78; 90°: r = 0.12, p = 0.67), concentric (Fig 2E and 2G) and eccentric (Fig 2I and 2K) at 30°/s and 60°/s. No significant relationships were evident between NH torque at break point angle and peak MVC torque in each contraction mode (Fig 2B, 2D, 2F, 2H, 2J and 2L).

**Fig 3:**
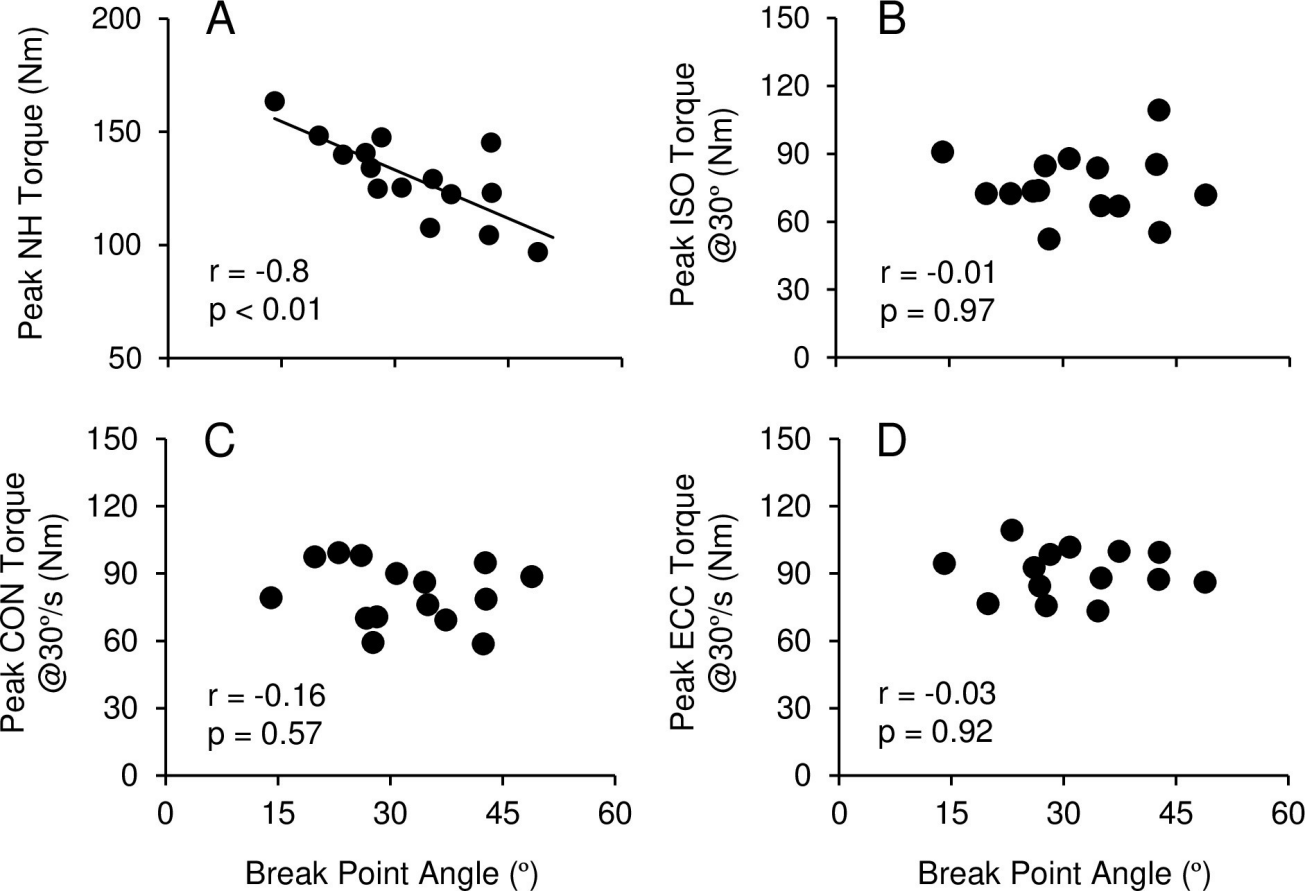
Correlations between break point angle in Nordic hamstring and peak Nordic hamstring torque (A), peak torque in maximal voluntary isometric contraction at 30° knee flexion (B), and concentric (C) and eccentric contraction at 30°/s (D) among 16 study participants. r and p values of Pearson correlation coefficient are shown in each figure.

As shown in Fig 3A, the break point angle in NH and peak NH torque were significantly correlated (r = −0.8, p<0.01). However, the break point angle in NH did not correlate with peak MVC torque in isometric knee flexion at 30° (Fig 3B) and other angles (90°: r = −0.03, p = 0.91; 60°: r = 0.14, p = 0.63; 45°: r = −0.06, p = 0.84). No significant correlations were also evident between the break point angle in NH and MVC concentric torque at 30°/s (Fig 3C) and 60°/s (r = −0.28, p = 0.31), as well as MVC eccentric torque at 30°/s (Fig 3D) and 60°/s (r = −0.13, p = 0.64). However, the break point angle in NH was significantly correlated with the angle at peak torque in eccentric knee flexion at 30°/s (r = 0.65, p<0.01) and 60°/s (r = 0.51, p = 0.045), but not with the angle at peak torque in concentric knee flexion at 30°/s (r = 0.2, p = 0.45) and 60°/s (r = 0.29, p = 0.25).

## Discussion

The present study found moderate relative reliability of the peak NH force, peak NH torque and angle at peak force measures; however, the break point angle, force and torque at break point and in NH showed large variabilities between attempts. The most important finding was that no significant correlations between the peak NH torque and the peak MVC torque in isometric, concentric, and eccentric knee flexion measures by the isokinetic dynamometer were evident, but a significant correlation (r = −0.8) was observed between the peak NH torque and the break point angle during NH. These results did not support the hypothesis that isometric and eccentric knee flexion torque would be significantly correlated with the NH strength.

As shown in Table 1, moderate relative reliability of the peak NH force and torque measures in which the speed of NH movement was approximately 30°/s was observed in the present study (ICC = 0.82–0.83, CV = 2.7%). This was similar to the previous study in which the test-retest reliability of the NH torque measure at 30°/s was examined using 25 healthy male student athletes (ICC = 0.85, CV = 7.4%) [16]. Wiesinger et al. [16] stated that the test-retest reliability of NH torque measures was higher (ICC = 0.94, CV = 5.4%) at the slower speed close to 15°/s. In the present study, the angle at peak force was moderately reliable (ICC = 0.86, CV = 8.3%), but the reliability of the break point angle (ICC = 0.54, CV = 8.4%) was not necessarily high. This was in line with the study by Wiesinger et al. [16] reporting that ICC was 0.54–0.58 for the reliability of the angle at peak torque. The present study used the highest NH force trial for the further analyses, based on the previous studies [15, 16].

When comparing to the previous studies in which NH force (361.2 N) [10] and NH torque (143 Nm) [16] at 30°/s was measured for sub-elite male athletes, the NH force in the present study (NH force: 325.6 N, NH torque: 130 Nm) appeared to be smaller, but larger than that of other studies (average: 298.6–301 N) in which professional male football players or professional and sub-elite rugby players were tested [11, 15]. It should be noted that the device to measure NH strength in the present study was not the same as that used in the previous studies. The angle at peak force in the present study (average: 32.5°) was smaller than that in the previous study in which male student athletes were tested (average: 39.8°) [16]. It may be that the differences were due to the difference in participants and a device used for the NH strength measures. However, it seems likely that the NH strength measure system used in the present study worked similarly to that used in the previous studies.

The most important finding of the present study was that the NH torque did not correlate with the knee flexion MVC torque in isometric (r = −0.03–0.1), concentric (r = 0.28–0.49) and eccentric contraction (r = 0.24–0.3) measured by the isokinetic dynamometer (Fig 2). van Dyk et al. [15] also reported a poor correlation (r = 0.35) between NH force and eccentric knee flexion torque at 60°/s in a seated position. In contrast, Wiesinger et al. [16] showed a significant correlation (r = 0.51–0.58, p<0.01) between NH torque and isokinetic (30°/s) eccentric knee flexion MVC torque. The present study showed that the average lean forward angular velocity during NH (28.8 ± 6.4°/s) was close to 30°/s; however, it was not possible to maintain a constant knee joint angular velocity during NH (Fig 1C). It was observed that the angular velocity was low during NH, which appeared to indicate that the knee extensors were performing isometric than eccentric contraction. Thus, it is possible that the NH strength is not necessarily a representative of eccentric strength of the knee flexors. It is important to note that NH was performed without securing the hip joint and trunk, but these joints were secured during the MVC torque measures on the isokinetic dynamometer. Thus, the hip extension and trunk extension muscles are likely to be involved during the NH. In fact, Sarabon et al. [23] reported that a half of the knee joint torque was exerted by the hip joint during NH. Bourne et al. [24] showed that a hip extension exercise training, in which the hip joint was extended while the knee joint was in extension, increased NH force. Timmins et al. [25] demonstrated that a hip-dominant flywheel exercise intervention increased the NH force similar to NH exercise intervention. These suggest that the hip extension (trunk extension) strength also affects the NH strength, but this should be confirmed in future studies.

To the best of our knowledge, this was the first study to examine the relationship between the NH strength and the break point angle. The present study showed that the break point angle was significantly correlated with the NH torque such that the smaller the angle (the greater the knee joint extension), the larger the torque (Fig 3A). The break point angle is considered to represent the ability to control the falling forward movement during NH. Therefore, the individuals who could lean forward more in NH (the smaller the break point angle) were able to exert greater NH strength. In future studies, the relationship between biomechanical characteristics of NH and NH strength should be investigated. It should be noted that the break point angle was significantly correlated with the angle at peak torque in eccentric knee flexion. This indicates that exerting peak eccentric knee flexion torque at longer muscle lengths make it possible to lean forward more during NH. Exerting higher peak eccentric knee flexion torque at longer muscle lengths has been considered a factor in decreasing the hamstring strain injury risk [26, 27]. Therefore, it may be possible to predict the risk of hamstring strain injury by the break point angle during NH. However, peak MVC torque in isometric, concentric, and eccentric contraction were not correlated with the break point angle (Fig 3B–3D). This suggests that not only the knee flexor strength but also other factors play a role in leaning forward during NH. Previous studies reported large muscle activity of internal and external oblique [28] or erector spine [23, 28] during NH. These trunk muscles likely keep the upper body straight during NH to stabilize the pelvic tilt and back. Recent studies suggested that trunk muscles’ function might be related to an incidence of hamstring strain injury [29, 30]. For example, Schuermans et al. [29] investigated the association between lower limb and trunk kinematics in sprinting and hamstring injury in male soccer players, and found that the players who had a hamstring strain injury showed higher levels of anterior pelvic tilting and thoracic side bending to support leg throughout the front swing phase of sprinting when compared with non-injured players. They also reported that injured players exerted lower trunk muscles activity during the back swing phase of sprinting and lower gluteal muscle activity during the front swing phase of sprinting than non-injured players [30]. Accordingly, activity of lumbopelvic muscles during NH appears to be an important factor affecting the NH strength.

Conventionally, an isokinetic dynamometer has been used to predict the hamstring strain injury risk from the knee flexors strength. In contrast, Green et al. [8] demonstrated that the knee flexion torque measured by an isokinetic dynamometer could not predict hamstring strain injuries well enough. The present study also showed no correlation between the isokinetic knee flexion torque and the NH strength (Fig 2). During the late-swing phase of sprinting that is considered to be a hamstring strain injury risk phase, the hamstring performs concentric contraction to extend a hip joint and eccentric contraction to absorb force in knee extension movement to decelerate the momentum [6, 7]. Considering these joint movement in the sprinting, NH strength is thought to be more useful measurement for predicting the hamstring strain injury risk than the knee flexion torque measures. van den Tillaar et al. [31] showed that knee and hip joint angles at peak hamstring EMG during NH were similar to respective joint angles during a sprinting. This supports the importance of NH strength as a predictor of hamstring strain injury. Bautista et al. [13] in their recent review paper documented that NH training was effective for increasing the NH strength, and preventing hamstring strain injuries. It is interesting to investigate the relationship between changes in NH strength and changes in knee flexion torque assessed by an isokinetic dynamometer. It should be noted that NH exercise training is not necessary highly effective for preventing hamstring strain injuries [12]. One of the reasons is that hamstring strain injuries are multifactorial [2]. Since the ability to control the core stability is also involved in the NH [23, 28], the core stability may affect the trainability of NH training. It is also important to note that NH’s hip and knee joint movements are much slower than those in sprinting and other movements inducing hamstring strain injuries [32]. Hence, NH exercise training may not be highly specific for movements in sports, which may be a reason why hamstring strain injuries cannot be prevented by NH exercise training.

The knee flexors strength measures by the isokinetic dynamometer in the present study (Table 2) were comparable to those reported in the previous studies in which young men were tested in a prone position [33, 34]. It should be noted that many of the previous studies [9, 15, 35] measured the knee flexion torque by an isokinetic dynamometer in a seated position. Findley et al. [34] reported the isokinetic concentric knee flexion torque measured in a seated position (99.2 Nm) was significantly larger than that in a prone position (87.1 Nm). Ayala et al. [33] stated that measuring the knee flexion torque in a prone position was functionally more relevant to predict the risk of hamstring strain injury, because the prone position simulates the hip joint angle, and the knee flexor and extensor muscle length-tension relationships in the late swing and early contact phase of sprinting when most athletes develop musculoskeletal lower limb injuries. Thus, the present study used the prone position to measure the knee flexor strength.

It has been reported that MVC torque of the knee flexors is greater in eccentric than isometric (18%) and concentric contraction (25%), and in isometric than concentric contraction (5%) [35]. In the present study, eccentric knee flexion MVC torque was 11–16% greater than isometric and concentric knee flexion MVC torque, but isometric knee flexion MVC torque was 7% smaller on average than concentric knee flexion MVC torque (Table 2). The angle at peak torque in the concentric knee flexion was 19.5–21.5° at an angular velocity of 30°/s and 60°/s (Table 2). A previous study reported that the angle at peak torque during concentric knee flexion in the prone position at an angular velocity of 60°/s was 11.4–16.0°[33]. These suggest that the optimal angle of the knee flexion torque in the prone position is around 10–20°. It should be noted that the testing angles of the isometric knee flexion torque measure in the present study was 30°, 45°, 60°, and 90° knee flexion. Thus, the lower isometric than concentric knee flexion torque in the present study may be due to the choice of the knee joint angle.

Limitations in this study should be addressed. We recruited university students with habitual resistance training with no previous hamstring strain injuries in the present study; therefore, the findings of this study may not be applied to athletes. It is interesting to compare the relationship between the NH strength and the knee flexors strength assessed by an isokinetic dynamometer for the previously injured and non-injured legs. In NH, its success or failure should be judged by setting the failure criterion. A previous study used the hip joint angle as a criterion of failure such that the NH trials in which the hip flexion exceeded 20° at any time point were discarded [16]. However, the present study defined the ability to control falling forward during NH based on the knee joint angular velocity. Nevertheless, the ability to stabilize the hip joint (trunk) in an extended position throughout the movement must be related to the operation of NH [23]. In addition, we did not evaluate the electromyography of muscles that contract during NH. Therefore, it cannot be denied that individual differences in the activity of hip and trunk muscles during NH affected NH strength. Future studies should discuss the relationship between the hip and trunk muscle activity and NH strength.

## Conclusion

The present study revealed that peak NH torque was not associated with the peak MVC torque in isometric, concentric, and eccentric knee flexion. However, a significant correlation was found between NH torque and the break point angle during NH, but the break point angle did not correlate with peak MVC knee flexion torque in isometric, concentric and eccentric contractions. It was concluded that NH strength did not relate to the knee flexion torque in a prone position but related to the ability to control the falling forward during NH.

## Perspectives

NH strength is not correlated with the MVC knee flexion torque in isometric, and isokinetic concentric and eccentric contractions, suggesting that NH strength and knee flexors strength measures are different. It has been documented that knee flexion torque testing may not be best suited to predict the future hamstring strain injury [8]. In contrast, NH strength measure has been shown to predict future hamstring strain injury risks, because the lower limb joint movements and hamstring muscle activity in the NH exercise resemble those in sprinting in which hamstring strain injuries are typically induced [23, 31]. Hence, coaches, athletic trainers, and clinicians should consider using the NH strength as a predictive measure for hamstring strain injury risks and as a criterion of return to play. The present study showed that the individuals who could lean forward more in the NH could exert greater NH strength. Therefore, a hamstring strain injury risk can be estimated on the field by assessing the break point angle during NH. The break point angle can be evaluated reliably by a smartphone-based application [36]. It may be also important to consider different movement velocities in the NH exercise training including faster velocity movements resembling the movements in sports.

## Supporting information

S1 Dataset (XLSX)
